# Including oxygen supplement in the early warning score: a prediction study comparing TOKS, modified TOKS and NEWS in a cohort of emergency patients

**DOI:** 10.1186/s13049-020-00720-1

**Published:** 2020-04-10

**Authors:** Maj Juhl Skov, Jacob Dynesen, Marie K. Jessen, Janet Yde Liesanth, Julie Mackenhauer, Hans Kirkegaard

**Affiliations:** 1 Research Center for Emergency Medicine, Emergency Department and Department of Clinical Medicine, Aarhus University Hospital and Aarhus University, Palle Juul-Jensens Boulevard 99, J103, 8200 Aarhus N, Denmark; 2 Department of Emergency medicine, Aarhus University hospital, Aarhus, Denmark; 3 Danish Center for Clinical Health Services Research (DACS), Department of Clinical Medicine, Aalborg University, Aalborg, Denmark

**Keywords:** Early warning score, oxygen, emergency department, TOKS, National Early Warning Score.

## Abstract

**Background:**

Early warning scores (EWS) are widely used in emergency departments and on general wards to detect critical illness and deterioration. TOKS (“Tidlig Opsporing af Kritisk Sygdom”) is an early warning score used in Central Denmark Region to monitor hospitalized patients.

The objective of this study is to investigate whether inclusion of supplement in the TOKS algorithm (modified TOKS; mTOKS), would improve the ability to predict 7-day mortality. Secondarily, we compare the discriminatory ability between TOKS, mTOKS and the National Early Warning Score (NEWS).

**Methods:**

This is a prediction study including a cohort of adult patients who attended an emergency department in Central Denmark Region during a 3-month period in 2015. The discriminatory ability of TOKS, mTOKS and NEWS was evaluated by calculating the area under the receiver operating characteristics- curve (AUROC) with 7-day mortality as outcome. mTOKS was defined by adding 2 points for oxygen supplement to the normal TOKS score.

**Results:**

18.853 patients were included. AUROC for TOKS: 0,78 (95%-CI: 0,76-0,81). AUROC for mTOKS: 0,81 (95 %-CI: 0,78-0,83). AUROC for NEWS: 0,83 (95%-CI: 0,80-0,85). The predictive ability of all three early warning scores are statistically significantly different from each other (*p*-value < 0,01).

**Conclusion:**

The discriminatory ability of TOKS improved statistically by including oxygen supplement. All models showed moderate to good discriminatory ability.

## Background

Early warning scores (EWS) are widely used in emergency departments and on general wards to detect critical illness and deterioration among hospitalized patients [1]. These systems are implemented to prevent serious adverse events e.g. cardiac arrest, admission to the intensive care unit (ICU) or death [1, 2].

Patient deterioration is often preceded by deviations in vital signs [3], and logically early warning scores are primarily based on these. For each vital parameter, cut-off points are set to determine the “normal range” and grade the extent of deviation. Points allocated for each vital sign add up to an aggregate EWS.

EWS is part of a rapid response system. A rapid response system consists of an afferent limb with staff measuring vital parameters and calculating a score, and an efferent limb consisting of an algorithm to trigger a call for a physician or medical emergency team [4].

Several EWS have been developed, implemented and adjusted worldwide [5]. In Denmark, the five regions use different locally established scores [6–8]. The score used in Central Region Denmark is named TOKS (Table 1) and was developed locally by a group of doctors and nurses. A guideline was released in 2010 [7]. TOKS was established by consensus decisions and has not been validated in an emergency setting. TOKS differs from other EWS by the selected cut-off points, and by not including oxygen supplement, despite high discriminatory performance of other scores including oxygen [2].

**Table 1:** EWS in Central Denmark Region [TOKS]

Vital signs	3	2	1	0	1	2	3
SysBP (mm Hg)	< 70	≥70 - < 80	≥80 - < 100	≥100 - < 200		≥200	
HR (beats pr. min)		< 40	≥40 - < 50	≥50 - < 90	≥90 - < 110	≥110 - < 130	≥130
Temp. (°C)	< 34	≥34 - < 36		≥36 - < 38	≥38 - < 39	≥39 - < 40	≥40
RR (breaths pr. min)	< 9		≥9 - < 12	≥12 - < 21		≥21 - < 25	≥25
SAT (%)	< 85	≥85 - < 90	≥90 - < 93	≥ 93			
LOC			Agitated	A	V	P	U

*sysBT* systolic blood pressure, *HR* Heart rate, *Temp*. Temperature, *RR* Respiration rate, *SAT* Saturation, *LOC* Level of consciousness, *A* Alert, *V* Voice, *P* Pain, *U* Unresponsive

The aim of this study was to compare TOKS with a modified TOKS (mTOKS) including 2 points for oxygen treatment. Moreover, we aim to compare the discriminatory ability between TOKS, mTOKS and the National Early Warning Score (NEWS) [9].

We hypothesize, that the ability of TOKS to discriminate patients at risk of dying could be improved, if two additional points are allocated to patients in supplemental oxygen therapy.

## Methods

### Study design and setting

This is a prediction study comparing three EWSs (TOKS, mTOKS and NEWS) in a paired design. All tests were performed on the same cohort of patients, registered in one of the five emergency departments (EDs) in Central Denmark Region (collectively ≈ 150.000 contacts/year) from March 1st until May 31th in 2015.

The EDs in Central Denmark Region serve a mixed rural-urban population of 1.3 million people and provide 24-h emergency care to all emergency patients except those transferred directly to catheterization laboratories (ST Elevation Myocardial Infarction patients), stroke units (thrombolysis candidates), women in labor or children with a medical emergency.

Health care in Denmark is tax-supported, subsidizing equal access to hospital treatment regardless of income. ED patients are either referred by the general practitioner or brought in by ambulance after an emergency call.

### Study population

ED patients under the age of 16 years, elective patients, registered healthy companions and patients without a Danish social security number, where deemed ineligible for this study. If a patient attends the ED more than once in the inclusion period, only the first hospital course was included.

We calculated an EWS from the first full set of vital signs registered under one time stamp. Patients who did not have a full set of vital signs within 4 h of their hospital course, were excluded from further analysis.

### Early warning scores

We primarily examine the EWS named TOKS in this study (Table 1). The score is used in Central Denmark Region for continuous monitoring of adult inpatients after triage in EDs and on regular wards. The score is based on systolic blood pressure, saturation, respiratory rate, heart rate, temperature and level of consciousness quantified using the AVPU scale (A = alert, V = responsive to vocals, P = responsive to pain), U = unresponsive) (Table 1) combined with an extra point for being agitated. The maximum possible TOKS is 18. TOKS is supplemented by a decision-making tool, guiding how often patients should be scored and whether urgent assessment by a junior or senior doctor is needed (supplementary Table 1).

When triaging patients in EDs in Central Denmark Region, all vital signs used in TOKS are measured, however Glasgow Coma Scale (GCS) is used instead of AVPU. In order to make triage measurements applicable for calculating initial TOKS score, GCS was converted into AVPU (GCS 15 = A, GCS 14 = V, GCS 9–13 = P, GCS ≤ 8 = U) in accordance with other studies [2, 10].

For every patient, we also calculated a score called modified TOKS (mTOKS). mTOKS uses the same cut-offs and weightings as TOKS but included two points for oxygen supplement in addition. The maximum possible score for mTOKS is 20. The allocation of 2 points was arbitrary but seemed reasonable to the research group. The algorithm for NEWS is shown in supplementary Table 2. The maximum possible NEWS is 20.

### Mortality and comorbidity

The outcome was death within seven calendar days, with day zero being the first day attending the ED. 7-day mortality was preferred over a shorter follow-up in order to secure enough cases of death and avoid statistical underpower. Charlson comorbidity index (CCI) was based on ICD-10 codes assigned to patients from 1 January 2004 until 1 week before the included hospital course and calculated based on the method suggested by Thygesen et al. [11, 12].

### Data source

Information on ED patients was retrieved from the regional patient data warehouse, which contains information about all patients’ hospital course, measured vital signs, diagnosis codes from present and previous hospitalizations and date of death. All clinical data was prospectively documented by the clinical staff in the electronic medical chart is stored in the data warehouse.

Data are registered under the unique personal social security number assigned to all Danish citizens at birth or upon immigration.

### Statistics

Continuous and categorical data are presented as medians (interquartile range (IQR)) and numbers (%), respectively. We used the area under the receiver operator characteristics curve (AUROC curve) to evaluate the ability of the original TOKS, mTOKS and NEWS to discriminate between those who die within 7 days and those who survive [13]. Each analysis was constructed by plotting the false positive rate (1-specificity) on the x-axis against the true positive rate (sensitivity) for each decision criterion for a prediction between 0 and 100%. An AUROC of 0.5, indicates that the model was no better than chance at predicting mortality. Using an algorithm for comparing AUROC-curves with paired data [14], we tested if the AUROCs were significantly different using a Chi-square test with a pre-set significance level of 0,05. STATA/SE 14.2 (StataCorp LP, College Station, TX, USA) was used for the analysis.

## Results

### Selection process and patient demographics

**Fig. 1:**
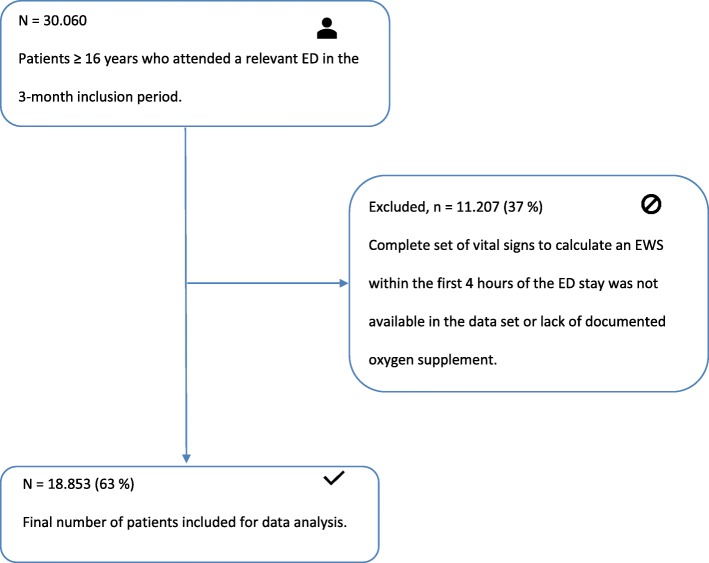
Selection process

A total of 30.060 patients ≥16 years with an emergency contact to one of the five sites in the 3-month inclusion period were identified. 11.207 (37%) of these patients were excluded because they did not have a complete set of vital signs within the first 4 h of their ED stay. 270 (2%) of these patients were excluded solely due to lack of documented oxygen supplement. 18.853 (63%) patients were included in the final analysis of the data set with at least one complete set of vital signs within 4 h of the ED stay (Fig. 1). Table 2 shows patient characteristics. Supplementary figure 1 shows the distribution of TOKS, mTOKS and NEWS in the study population. The figure also shows the mortality related to a specific EWS.

**Table 2:** Patient characteristics

	Patients enrolled in the study *N* = 18.853	Patients excluded from the study *N* = 11.207
Female gender n (%)	9.765 (52%)	5.975 (53%)
Median age in years and IQR	61 [41;76]	41 [25;60] *
7-day mortality (%)	349 (2%)	23 (0,2%) *
CCI (n %)		
CCI 0	11.600 (62%)	9.476 (85%)
CCI 1–2	4.652 (25%)	1.293 (12%)
CCI 3+	2.601 (14%)	438 (4%)
Number of patients stratified by hospital		
Aarhus	5.327 (28%)	3.791 (34%)
Horsens	2.557 (14%)	2.340 (21%)
Viborg	3.475 (18%)	1.794 (16%)
Randers	3.837 (20%)	2.146 (19%)
Hospitalsenhed Vest	3.657 (19%)	1.136 (10%)

*CCI* Charlson comorbidity index, *N* number of patients

*Significantly different from the enrolled patients, *p*-value < 0,001

### ROC curves

**Fig. 2:**
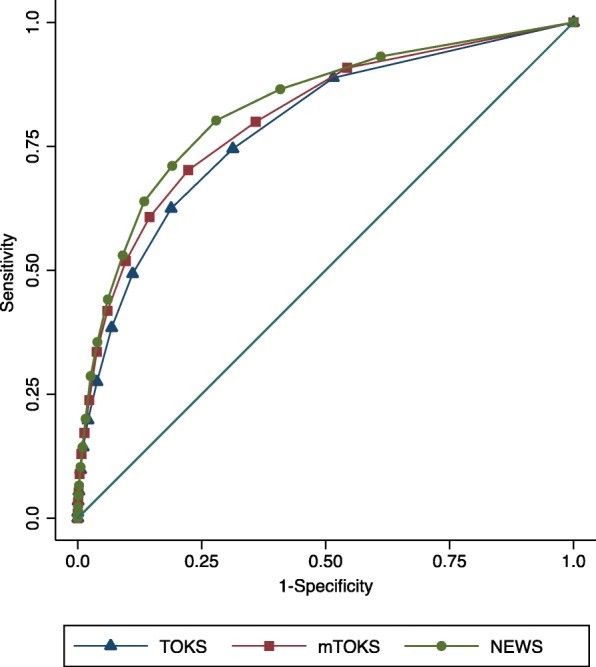
Area Under the Receiver Operating Characteristics (AUROC) curves for TOKS, mTOKS and NEWS

The ROC-curves for TOKS, mTOKS and NEWS are shown in Fig. 2. AUROC (TOKS): 0,79 with 95%-CI [0,76 – 0,81]. AUROC (mTOKS): 0,81 with a 95% CI [0,78 – 0,83]. AUROC (NEWS): 0,83 with a 95%-CI [0.80–0.85].

The AUROC for TOKS indicate reasonable discrimination, while both AUROCs for mTOKS and NEWS indicate a good discrimination, however NEWS performs better than TOKS. The predictive ability of all three early warning scores are statistically significantly different from each other (*p*-value < 0,01).

## Discussion

In this study of emergency department patients, we found that including 2 points for oxygen supplement in TOKS improved the score’s ability to predict death within 7 days. This finding suggest that it could be beneficial to include oxygen therapy in TOKS. Furthermore, our study shows that NEWS had a statistically better discriminatory ability, when it comes to predicting 7-day mortality, than both TOKS and mTOKS respectively. However, all models showed moderate to good discriminatory ability.

Discussion and continuous improvement of tools to detect critical illness in hospitals is essential to ensure patient safety. In this study we chose to look at oxygen therapy, because this treatment influences other vital signs, altering the aggregate EWS. Oxygen therapy is used for patients with respiratory distress and helps decrease the respiratory rate, increase saturation thus reducing hypoxemia [15]. In the acute setting, oxygen therapy is merely a symptomatic treatment, which does not necessarily improve the underlying cause of respiratory distress. Because oxygen therapy might to some degree mask an underlying threatening condition, it could justify compensatory points. Furthermore, oxygen supplement could simply be prescribed based on a gut feeling before vital signs are measured.

Studies from United Kingdom (UK) have shown that the national early warning score (NEWS) has the best discriminatory ability of in hospital mortality, cardiac arrest and transfer to ICU when compared to 33 other early warning scores [2]. NEWS was developed empirically by a group consisting of doctors and nurses appointed by the Royal College of physicians in 2012 [9]. They first developed a forerunner called Vitalpack EWS (ViEWS) [1]. Pryterch et al. stated that ViEWS emerged by a “trial and error” approach, to gain the best AUROC discrimination of 24-h in-hospital mortality. This was obtained by adjusting the weightings and vary the ranges for each EWS component. Two points for oxygen supplement was included in the final version of NEWS, because it improved the overall discriminatory ability, as evident by an increase in AUROC. The clinical guideline on TOKS was published in 2010 [7]. NEWS was not yet developed at the time and other studies on EWS generally showed low sensitivity [16, 17]. Since no specific scoring system seemed optimal, TOKS was developed based on available international literature combined with consensus decisions.

We excluded 37% of the patients because they did not have a full set of vital parameters to calculate TOKS, mTOKS and NEWS. Many patients attended the EDs in Central Region Denmark due to minor injuries e.g. a sprained ankle or a broken finger. These patients will, according to the triage algorithm, automatically not have vital signs measured (supplementary Table 1). Patients may also be excluded due to inadequate documentation of vital signs. A Danish study from Petersen et al. found that inadequate documentation is often due to lack of time [18]. Further, since oxygen supplement is not a part of TOKS, it is questionable how well this parameter is documented. However, we found that a minority of patients were excluded solely because they lacked documentation on oxygen supplement, indicating documentation of oxygen supplement to be a part of the daily routine.

The outcome was death within 7 days after the first set of vital signs were measured. We could have chosen 24- or 48-h mortality. These short-term endpoints might have a stronger correlation to the EWS score, thus presumably improving the discriminatory ability of all three early warning scores. Thus, choosing 7-day mortality could underestimate the discriminatory ability.

AUROC may be optimistic, since no data splitting was conducted. However, using univariable models we do not expect the true AUROC to differ significantly.

Many different endpoints have been used in the search of the best EWS. e.g. Cardiac arrest, transfer to ICU, death or in-hospital mortality. Concerns about the value of these endpoints have been discussed. Critics say that one should be careful not to optimize EWS only to find patients who cannot be saved, even with timely and correct care [19]. Thus, it is important that we develop an EWS that can predict critical illness within patients whose condition can be turned around.

We were not able to exclude patients with “do not resuscitate” orders. Unfortunately, these patients may fall into the group of patients who cannot be saved and are therefore more likely to die. This could underestimate the discriminatory ability of all three early warning scores. In future studies this group should be eliminated.

We chose to convert the GCS into AVPU in order to use the vital parameters from triage. The applied conversion algorithm has not been validated. TOKS allocates one point to the agitated person, but GCS does not account for agitation. This means that some agitated patients will get 0 points because level of consciousness was assessed using GCS. This could underestimate the discriminatory ability of TOKS and mTOKS if agitation has a significant correlation to death.

This study has multiple strengths. We used a large database including patients from five different hospitals in Central Denmark Region. Data on vital signs were collected as a part of the daily routine. This makes the study population representative to the Danish population in general. In future studies it could be interesting to compare all the EWS systems in Denmark, test other cut-off values for each vital parameter and incorporate other endpoints than death.

## Conclusion

Inclusion of oxygen supplement in the early warning score TOKS improves the ability to discriminate patients at risk of death within 7 days. The discriminatory ability of NEWS is statistically superior to both TOKS and mTOKS when it comes to discriminating 7-day mortality, but all models show moderate to good discriminatory ability.

## Supplementary information

**Additional file 1 **: **Table S1** Decision-making tool associated with TOKS

**Additional file 2 **: **Table S2** National Early Warning Score – NEWS

**Additional file 3 **: **Figure S1** Distribution of EWS and mortality in the study population

## Data Availability

Data for the study has been retrieved from the Danish National Patient Register and our Regional data Warehouse. Restrictions apply to the availability and we will therefore not be able to leave these publicly available.

## Abbreviations

EWS: Early Warning Score
TOKS: [Tidlig Opsporing af Kritisk Sygdom]
mTOKS: Modified “TOKS”
NEWS: National Early Warning Score
AUROC: Area Under the Receiver Operating Characteristics
ICU: Intensive Care Unit
LOC: Level of Consciousness
ED: Emergency Department
IQR: Interquartile Range
GCS: Glasgow Coma Score
AVPU: Alert-vocal-pain-unresponsive
