# Supplementary material for: Including oxygen supplement in the early warning score: a prediction study comparing TOKS, modified TOKS and NEWS in a cohort of emergency patients
Source: Scand J Trauma Resusc Emerg Med. 2020 Apr 10;28:26. doi: 10.1186/s13049-020-00720-1 (PMC7147010; doi:10.1186/s13049-020-00720-1)
Supplement: Supplementary file 1 — Additional file 1 : Table S1 Decision-making tool associated with TOKS [file 13049_2020_720_MOESM1_ESM.docx]

**Supplementary table 1** Decision-making tool associated with TOKS

| **Decision-making tool** | | |
| --- | --- | --- |
| **Score** | **Observation frequency and allowed score** | **Action** |
| 0 | Vital signs are measured 1 time every 24 hours | None |
| 1 | Vital signs are measured 3 times every 24 hours | Nurses optimize vital signs |
| 2 | Vital signs are measured again after 1 hour | Nurses optimize vital signs |
| 3-4 or a single score ≥ 2 | Doctor prescribes observation frequency and determines an individual threshold score that should lead to renewed doctor evaluation. | Nurses optimize vital signs and  contacts a junior doctor to evaluate the patient |
| ≥ 5 | Doctor prescribes observation frequency and determines an individual threshold score that should lead to renewed doctor evaluation. | Nurses optimize vital signs and contacts a senior doctor to evaluate the patient |
