# Supplementary material for: Including oxygen supplement in the early warning score: a prediction study comparing TOKS, modified TOKS and NEWS in a cohort of emergency patients
Source: Scand J Trauma Resusc Emerg Med. 2020 Apr 10;28:26. doi: 10.1186/s13049-020-00720-1 (PMC7147010; doi:10.1186/s13049-020-00720-1)
Supplement: Supplementary file 2 — Additional file 2 : Table S2 National Early Warning Score – NEWS [file 13049_2020_720_MOESM2_ESM.docx]

**Supplementary table 2** National Early Warning Score – NEWS

**Table 2** – National Early Warning Score

| Vital signs | 3 | 2 | 1 | 0 | 1 | 2 | 3 |
| --- | --- | --- | --- | --- | --- | --- | --- |
| Systolic BP (mm Hg) | <90 | 91 - 100 | 101-110 | 111-219 |  | ≥220 |  |
| HR (beats pr. min) | <40 |  | 41 - 50 | 51 - 90 | 91 - 110 | 111 - 130 | ≥131 |
| Temperature (°C) | <35.0 |  | 35.1 – 36.0 | 36.1 – 38.0 | 38.1 – 39.0 | ≥39.1 |  |
| RR (breaths pr. min) | ≤ 8 |  | 9 - 11 | 12 - 20 |  | 21 - 24 | ≥25 |
| Sat. (%) | <91 | 92 - 93 | 94 - 95 | ≥ 96 |  |  |  |
| LOC |  |  |  | A |  |  | V, P or U |
| Any supplemental oxygen |  | Yes |  | No |  |  |  |

*BP = Blood pressure, HR = Heart rate, RR = Respiration rate, Sat. = Saturation, LOC = level of consciousness, A = alert, V = voice, P = pain, U = unresponsive.
