# Supplementary material for: Including oxygen supplement in the early warning score: a prediction study comparing TOKS, modified TOKS and NEWS in a cohort of emergency patients
Source: Scand J Trauma Resusc Emerg Med. 2020 Apr 10;28:26. doi: 10.1186/s13049-020-00720-1 (PMC7147010; doi:10.1186/s13049-020-00720-1)
Supplement: Supplementary file 3 — Additional file 3 : Figure S1 Distribution of EWS and mortality in the study population [file 13049_2020_720_MOESM3_ESM.docx]

**Supplementary figure 1** Distribution of EWS and mortality in the study population.
